# Development and validation of two online dynamic nomograms for patients with non‐distant metastatic cutaneous melanoma based on surgical approaches

**DOI:** 10.1002/cam4.6448

**Published:** 2023-08-18

**Authors:** Shiqi Wang, Yuedong Chen, Jiawei Sun, Ran Mo, Qian Tan

**Affiliations:** ^1^ Department of Burns and Plastic Surgery Affiliated Drum Tower Hospital, Medical School of Nanjing University Nanjing People's Republic of China; ^2^ Department of Burns and Plastic Surgery Affiliated Jinling Hospital, Medical School of Nanjing University Nanjing People's Republic of China; ^3^ Diabetic Foot Center Nanjing Junxie Hospital Nanjing People's Republic of China

**Keywords:** amputation, cutaneous melanoma, excision margins, nomogram, prognosis, surgical approaches

## Abstract

**Background and Objectives:**

Surgery is an essential treatment for non‐distant metastatic cutaneous melanoma (NMCM). We aim to construct and validate prognostic nomograms based on surgical approaches and the clinicopathological characteristics of NMCM patients.

**Methods:**

Data of patients diagnosed with cutaneous melanoma from 2004 to 2015 were identified from the Surveillance, Epidemiology, and End Results (SEER) database. Two online nomograms were constructed to predict the 3, 5‐year melanoma‐specific survival (MSS) for NMCM patients based on the surgical approaches. These nomograms were evaluated by the dynamic Harrell's concordance index (C‐index), decision curve analysis and clinical impact curve. Both internal and external data verification were conducted.

**Results:**

A total of 14,091 NMCM cases were included in this study. The C‐index of the nomograms for the excisional surgery group and amputation group were 0.818 and 0.806, respectively, and 0.763 and 0.731, respectively, in our hospital data validation. After internal and bootstrap verification, our two nomograms showed good accuracy and practicality.

**Conclusion:**

NMCM patients exhibited equal survival rates independent of resection margin size, while those who needed amputation had worse survival rates. We generated two online nomograms distinguished by surgical approach to predict NMCM patient survival based on clinicopathological characteristics.

## Abstract 2

Our study, based on the Cox survival analysis of large‐amount SEER data, provides evidence that surgical margins 1–2 cm or >2 cm have no significant impact on survival in NMCM patients, while suggesting using amputation for CM with more caution. We developed two simple‐to‐use nomograms and corresponding online web calculators for the excisional surgery and amputation groups.

## 1 INTRODUCTION

Cutaneous melanoma (CM) is a type of cancer originating from neural crest cells; it accounts for 1.7% of all newly diagnosed malignant cancers worldwide and causes approximately 55,500 deaths annually.^1^ Due to higher age‐specific rates in the elderly, as the aging population increases, so will the incidence of melanoma, making it the deadliest type of skin cancer.^2^

Surgery is an indispensable part of melanoma treatment without distant metastasis. For wide local surgery, the choice of surgical margin has been controversial. The National Comprehensive Cancer Network and the American Academy of Dermatology advise that for a tumor more than 2 mm thick, the surgical margin should be 2 cm.^3^, ^4^ Recent randomized cohort studies have reported that survival is unaffected regardless of whether the surgical margin is 0–2 cm or 3–4 cm.^5^, ^6^ Amputation, as a more aggressive eradication surgery for non‐distant metastatic cutaneous melanoma (NMCM), also need extraordinary consideration. It is uncertain if the correlation between amputation and the following low long‐term survival is a result of the patient's existing poor health or the hemodynamic and psychological effects of amputation itself.^7^, ^8^ However, due to the lack of sufficient data at single medical centers, there are few studies on the prognosis of surgical approaches, especially amputation‐related CM. In order to understand the relationship between surgical approaches and survival rates, and to provide clinicians with more accurate prediction models for appropriate clinical decision‐making, we performed the prognostic analysis of different surgery approach groups with sufficient data from the Surveillance, Epidemiology, and End Results (SEER) Program of the National Cancer Institute. Meanwhile, we developed and validated survival prediction models visualized as online nomograms—a clinical decision‐making tool with a more intuitive and user‐friendly interface and is widely used in medicine. The accuracy of the prediction models was verified by internal verification based on random cohort and external verification based on our hospital data and compared with the American Joint Committee on Cancer (AJCC) 8th staging system.

## 2 MATERIALS AND METHODS

### 2.1 Data source

**FIGURE 1:**
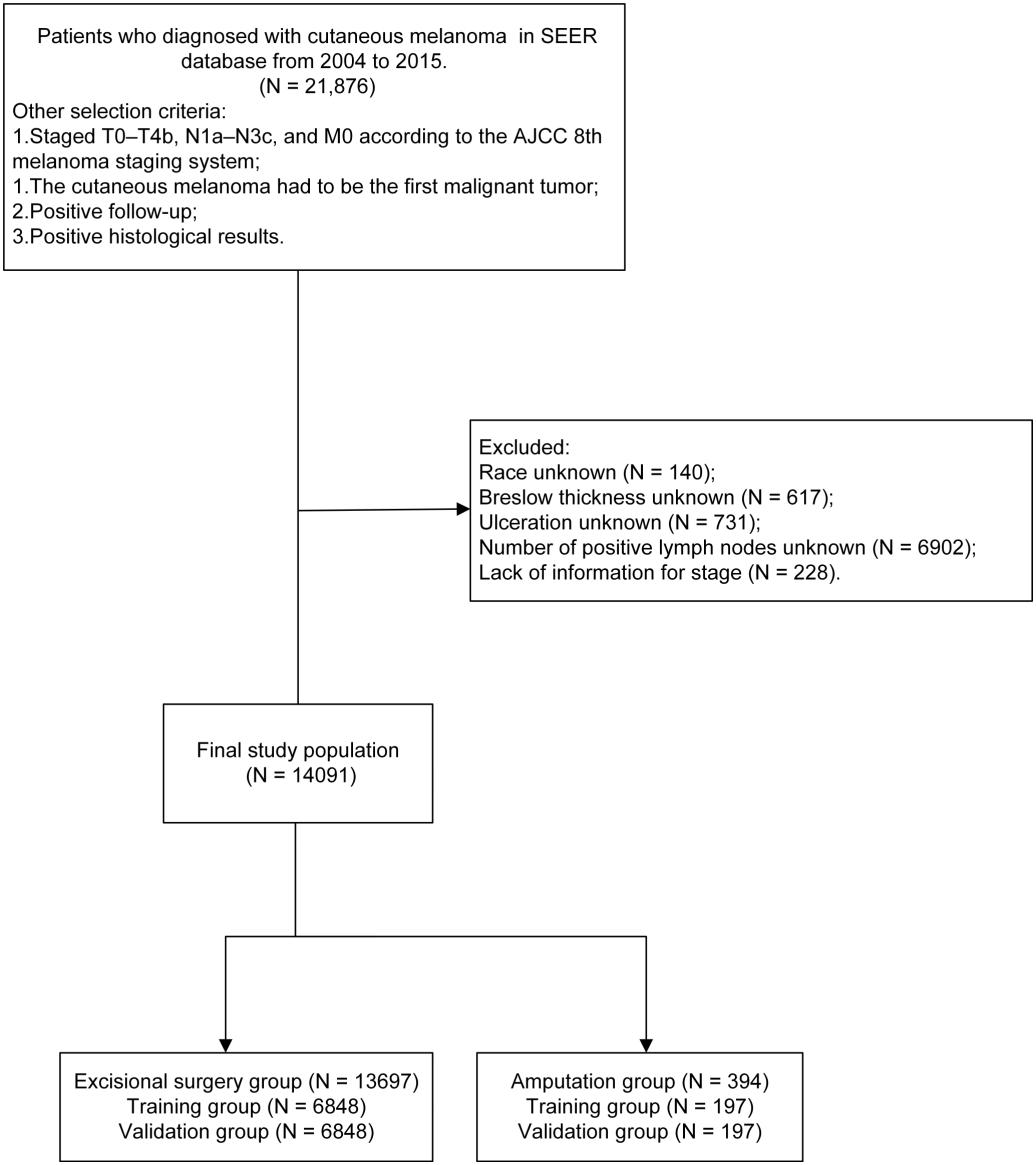
Flow chart for screening patient data from SEER database.

Patient data were acquired from the American National Cancer Institute's SEER database (http://seer.cancer.gov/seerstat/). The process of screening patient data is shown in Figure 1. Data were collected from 21,876 patients who were diagnosed with CM between 2004 and 2015. The external verification data were collected from the Affiliated Drum Tower Hospital, Medical School of Nanjing University from 2010 to 2020, and the characteristic variables and screening methods were the same as those of the SEER cohort.

The factors we collected included age, gender, race, AJCC 8th N stage, T stage, number of PLNs (number of positive lymph nodes, which was counted during sentinel node biopsy and lymph nodes removed), primary tumor location, Breslow thickness, ulceration, pathological subtype, surgical approach, and survival months. Because the AJCC 8th N stage and T stage were determined according to the number of positive lymph nodes and Breslow thickness, respectively, we excluded these two variables to avoid repeated factors to ensure the simplicity and ease of use of the model. According to the guidelines, the surgical procedures were divided into three types: 1–2 cm, >2 cm, and amputation.^3^, ^4^ The end‐point events were melanoma‐specific survival (MSS) or death. MSS time was defined as the time interval between initial diagnosis and death from melanoma.

This study was approved by the Ethics Committee of Nanjing Drum Tower Hospital, the Affiliated Hospital of Nanjing University Medical School (approval no. 2017‐175‐01).

### 2.2 Statistical analysis

Patient characteristics were summarized as the number of classified variables (%) and the means ± the standard deviation (SD) of continuous variables. Chi‐square tests were used to compare the grouping differences of characteristic variables under different surgical methods. The Cox proportional hazard regression analysis was used to explore the risk factors related to MSS. The hazard ratio (HR) and its 95% confidence interval (CI) were calculated. The survival function was estimated by Kaplan–Meier analysis, and the difference in MSS stratified by each covariable was evaluated by the log‐rank test. Based on the results of multivariate analysis, nomograms were established to predict the probability of 3‐ and 5‐year MSS in the excisional surgery and amputation group, respectively. The training group and the internal verification group were randomly split. Internal verification and bootstrap verification (performed by bootstrapping with 1000 resamples) were conducted after the model was established by the training group. The recognition ability of the nomograms was evaluated by a time‐dependent dynamic Harrell's concordance index (C‐index) and was compared with the AJCC 8th staging system. Decision curve analysis (DCA) and clinical influence curves were used to evaluate the clinical practicability of the two models. DCA quantifies net income under different threshold probabilities. In the clinical influence curve of the model, the solid blue line indicates the number of people who are classified as high risk by the model under each threshold probability, while the yellow dotted line is the number of true positive people under each threshold probability. A two‐tailed *p* < 0.05 was considered statistically significant. All statistical analyses and mapping were conducted using R 4.1.1 software (The R Foundation for Statistical Computing).

## 3 RESULTS

### 3.1 Baseline data

The process of screening patient data from SEER database is shown in Figure 1. Data from 21,876 patients were collected from 2004 to 2015 SEER database. We retained patients with melanoma stage M0, which means no distant metastases. Cases without enough information on race, ulceration, Breslow thickness, sentinel node biopsy, and AJCC 8th stage were excluded. Data from 14,091 patients remained, of which 10,162 (72.12%) underwent 1–2 cm margin excisional surgery, 3535 (25.09%) underwent >2 cm margin excisional surgery and 394 (2.8%) underwent amputation (Table 1).

**TABLE 1:** Baseline clinicopathologic characteristics according to different surgical approaches. AJCC, American Joint Committee on Cancer; MSS, melanoma‐specific survival.

	*n* (%)				
Variables	1–2 cm margin (*n* = 10,162, 72.12%)	>2 cm margin (*n* = 3535, 25.09%)	Amputation (*n* = 394, 2.80%)	Total (*n* = 14,091)	*p*‐value
Gender					0.067
Female	4236 (41.68)	1402 (39.66)	172 (43.65)	5810 (41.23)	
Male	5926 (58.32)	2133 (60.34)	222 (56.35)	8281 (58.77)	
Race					<0.001
White	9998 (98.39)	3458 (97.82)	344 (87.31)	13,800 (97.93)	
Black	54 (0.53)	27 (0.76)	20 (5.08)	101 (0.72)	
Others	110 (1.08)	50 (1.41)	30 (7.61)	190 (1.35)	
Age	56.59 ± 15.72	56.49 ± 15.85	62.23 ± 14.16	56.72 ± 15.74	
Age					<0.001
<18	74 (0.73)	35 (0.99)	1 (0.25)	110 (0.78)	
18–44	2128 (20.94)	721 (20.40)	49 (12.44)	2898 (20.57)	
45–54	2162 (21.28)	748 (21.16)	58 (14.73)	2968 (21.06)	
55–64	2487 (24.47)	911 (25.77)	103 (26.14)	3501 (24.85)	
65–74	1915 (18.84)	640 (18.10)	99 (25.13)	2654 (18.83)	
≥75	1396 (13.74)	480 (13.58)	84 (21.32)	1960 (13.90)	
Location					<0.001
Head and neck	1427 (14.04)	442 (12.50)	19 (4.8)	1888 (13.40)	
Trunk	3661 (36.03)	1480 (41.87)	3 (0.76)	5144 (36.51)	
Upper limb and shoulder	2872 (28.25)	859 (24.30)	125 (31.73)	3856 (27.36)	
Lower limb and hip	2188 (21.53)	743 (21.02)	246 (62.44)	3177 (22.55)	
Others	14 (0.14)	11 (0.31)	1 (0.25)	26 (0.18)	
Subtype of melanoma					<0.001
Nodular melanoma	1801 (17.72)	649 (18.36)	63 (15.99)	2513 (17.83)	
Lentigo maligna melanoma	210 (2.07)	62 (1.75)	2 (0.51)	274 (1.94)	
Superficial spreading melanoma	3274 (32.22)	1015(28.72)	36 (9.14)	4325 (30.69)	
Acral lentiginous melanoma	152 (1.50)	68 (1.92)	117 (29.70)	337 (2.39)	
Melanoma not specified	4063 (39.98)	1505 (42.57)	159 (40.36)	5727 (40.64)	
Others	662 (6.51)	236 (6.68)	17 (4.31)	915 (6.49)	
Lymph node count	1.00 ± 1.28	0.41 ± 1.40	1.10 ± 2.88	0.39 ± 1.38	
Lymph node count					<0.001
0	8280 (81.48)	2798 (79.15)	254 (64.47)	11,332 (80.42)	
1	1203 (11.83)	457 (12.93)	64 (16.24)	1724 (12.23)	
2–3	516 (5.08)	217 (6.14)	46 (11.68)	779 (5.53)	
4–5	83 (0.82)	30 (0.85)	12 (3.05)	125 (0.89)	
>5	80 (0.79)	33 (0.93)	18 (4.57)	131 (0.93)	
Ulceration					<0.001
No	7359 (72.42)	2484 (70.27)	137 (34.77)	9980 (70.83)	
Yes	2803 (27.58)	1051 (29.73)	257 (65.23)	4111 (29.17)	
Breslow thickness (Mean ± SD) (mm)	2.24 ± 2.03	2.40 ± 2.20	3.63 ± 2.72	2.32 ± 2.11	
Breslow thickness (mm)					<0.001
≤1.00	2661 (26.19)	1006 (28.46)	60 (15.23)	3727 (26.45)	
1.01–2.00	3810 (37.49)	1106 (31.29)	85 (21.57)	5001 (35.49)	
2.01–4.00	2321 (22.84)	822 (23.25)	98 (24.87)	3241 (23.00)	
>4.00	1370 (13.48)	601 (17.00)	151 (38.32)	2122 (15.06)	
AJCC 8th stage					<0.001
I	5202 (51.19)	1734 (49.05)	87 (22.08)	7023 (49.84)	
II	3033 (29.85)	1050 (29.70)	165 (41.88)	4248 (30.15)	
III	1023 (18.96)	1797 (21.24)	142 (36.04)	2820(20.01)	
MSS					<0.001
Alive	8871 (87.30)	3005 (85.01)	276 (70.05)	12,152 (86.24)	
Dead	1291 (12.70)	530 (14.99)	118 (29.95)	1939 (13.76)	

In the 1–2 cm margin excisional surgery and >2 cm margin excisional surgery groups, most were male, mainly white, and mostly between 18 and 64 years (66.89% and 67.43%, respectively). The most common location of the tumor was in the truck (36.03% and 41.87%, respectively). Except for uncertain melanoma, the most common pathological type was superficial spreading melanoma (32.22% and 28.72%, respectively). The probability of all negative lymph nodes was 81.48% and 79.15%, respectively. Moreover, most of the tumors did not have ulcers (72.42% and 70.27%, respectively), Breslow thickness was mainly ≤2.00 mm (63.68% and 59.75%, respectively), and AJCC 8th Stage I was the most common (51.19% and 49.05%, respectively).

Except for gender, the distribution of other characteristic variables in the amputation group differed from that in the excisional surgery group. Whites still accounted for the highest proportion of the race, but the proportion decreased (87.31%), while the age increased, mainly ≥55 years old (72.59%). The primary tumor location was concentrated in the lower limb and hip (62.44%). Except for melanoma not specified, the most common pathological type was acral lentiginous melanoma (ALM) (29.70%). Although the total negative probability of lymph nodes still accounted for the highest proportion, the proportion was decreased (64.47%). Most of the tumors had accompanying ulceration (65.23%). Breslow thickness was dominated by ≥2.01 mm, and AJCC 8th stages were mainly Stage II (41.88%) and Stage III (36.04%).

The data screening process in our hospital is demonstrated in Figure S1. A total of 102 NMCM patients' medical records were collected, and after screening, an external validation set was constructed from 83 patient cases with complete materials. Among them, 67 underwent excisional surgery, and 16 underwent amputation surgery. More details can be found in Table S1.

### 3.2 Cox proportional hazards regression analyses

We used the Cox proportional hazards regression analysis to compare the effect of different surgical approaches on survival. The results showed that after balancing factors including gender, age, location, ulceration, pathological subtypes, Breslow thickness, LN count and AJCC 8th stage, the surgical approach proved to be an independent influencing factor. Compared to with a 1–2 cm excision margin, >2 cm excision margin group had the similar survival rate (>2 cm surgical margin vs. 1–2 cm surgical margin: HR 1.00, 95% CI 0.90–1.10, *p* = 0.951) (Table 2), but amputation group had a significantly lower survival rate of NMCM (amputation vs. 1–2 cm surgical margin: HR 1.48, 95% CI 1.20–1.83, *p* < 0.001) (Table 2).

**TABLE 2:** Multivariate Cox regression analysis of surgery approach after balancing factors including gender, age, location, ulceration, pathological subtypes, Breslow thickness, LN count and AJCC 8th stage.

Variable	HR	95% CI	*p*‐Value
Surgery approach			
1–2 cm margin			
>2 cm margin	1.00	0.90–1.10	0.951
Amputation	1.48	1.20–1.83	<0.001

### 3.3 Predictors of survival

Given that Cox regression analysis showed a significant decrease in survival in the amputation group, we next explored the factors that impacted survival in the excisional surgery group (both 1–2 cm and >2 cm margins) and the amputation group, respectively.

In the excisional surgery group, after the characteristic variables were substituted into multivariate analysis and corrected other factors, the factors independently related to MSS included gender (male vs. female: HR 1.39, 95% CI 1.25–1.55), age (18–44 vs. <18: HR 2.55, 95% CI 1.05–6.20; 45–54 vs. <18: HR 3.76, 95% CI 1.55–9.12; 55–64 vs. <18: HR 4.38, 95% CI 1.81–10.60; 65–75 vs. <18: HR 5.59, 95% CI 2.31–13.52; ≥75 vs. <18: HR 7.75, 95% CI 3.20–18.77), pathological subtype (Superficial spreading melanoma vs. Nodular melanoma: HR 0.85, 95% CI 0.74–0.97), number of PLNs (single vs. none: HR 2.71, 95% CI 2.41–3.05; 2–3 vs. none: HR 3.88, 95% CI 3.38–4.47; 4–5 vs. none: HR 5.82, 95% CI 4.51–7.52; >5 vs. none: HR 8.71, 95% CI 6.74–11.27), ulceration (yes vs. no: HR 1.91, 95% CI 1.73–2.12), primary tumor location (trunk vs. head and neck: HR 0.81, 95% CI 0.71–0.92; upper limb and shoulder vs. head and neck: HR 0.64, 95% CI 0.55–0.74; lower limb and hip vs. head and neck. HR 0.60, 95% CI 0.51–0.71), Breslow thickness (mm) (1.01–2.00 vs. ≤1.00: HR 1.46, 95% CI 1.22–1.74; 2.01–4.00 vs. ≤1.00: HR 2.39, 95% CI 2.01–2.85; >4.00 vs. ≤1.00: HR 3.77, 95% CI 3.14–4.53) (Table 3). The selected factors were analyzed by MSS survival analysis, and the results are shown in Figures S2 and S3.

**TABLE 3:** Multivariate analysis of factors affecting melanoma‐specific survival.

	Excision Surgery (*N* = 13,697)			Amputation (*N* = 394)		
Variables	HR	95% CI	*p*‐value	HR	95% CI	*p*‐value
Gender			<0.001			0.025
Female	1			1		
Male	1.39	1.25–1.55		1.57	1.06–2.32	
Age						
<18	1					
18–44	2.55	1.05–6.20	0.038			
45–54	3.76	1.55–9.12	0.003			
55–64	4.38	1.81–10.60	0.001			
65–75	5.59	2.31–13.52	<0.001			
≥75	7.75	3.20–18.77	<0.001			
Location						
Head and neck	1					
Trunk	0.81	0.71–0.92	0.001			
Upper limb and shoulder	0.64	0.55–0.74	<0.001			
Lower limb and hip	0.60	0.51–0.71	<0.001			
Others	0.59	0.15–2.36	0.466			
Subtype of melanoma						
Nodular melanoma	1					
Lentigo maligna melanoma	1.01	0.70–1.44	0.979			
Superficial spreading melanoma	0.85	0.74–0.97	0.018			
Acral lentiginous melanoma	1.10	0.81–1.50	0.550			
Melanoma not specified	0.90	0.80–1.01	0.085			
Others	0.85	0.70–1.04	0.106			
Lymph node count						
0	1			1		
1	2.71	2.41–3.05	<0.001	3.51	2.17–5.66	<0.001
2–3	3.88	3.38–4.47	<0.001	6.40	3.90–10.48	<0.001
4–5	5.82	4.51–7.52	<0.001	3.92	1.52–10.15	0.005
>5	8.71	6.74–11.27	<0.001	6.97	3.46–14.00	<0.001
Ulceration						
No	1			1		
Yes	1.91	1.73–2.12	<0.001	1.35	0.82–2.22	0.245
Breslow thickness (mm)						
≤1.00	1			1		
1.01–2.00	1.46	1.22–1.74	<0.001	1.19	0.49–2.88	0.698
2.01–4.00	2.39	2.01–2.85	<0.001	1.70	0.74–3.91	0.212
>4.00	3.77	3.14–4.53	<0.001	3.27	1.51–7.08	0.003

In the amputation group, after the characteristic variables were substituted into the multivariate analysis and corrected for other factors, the factors independently related to MSS included gender (male vs. female: HR 1.57, 95% CI 1.06–2.32), number of PLNs (single vs. none: HR 3.51, 95% CI 2.17–5.66; 2–3 vs. none: HR 6.40, 95% CI 3.90–10.48; 4–5 vs. none: HR 3.92, 95% CI 1.52–10.15; >5 vs. none: HR 6.97, 95% CI 3.46–14.00), and Breslow thickness (mm) (>4.00 vs. ≤1.00: HR 3.27, 95% CI 1.51–7.08) (Table 2). After the inclusion of the characteristic ulceration variable, the C‐index of the prediction model rose from 0.801 to 0.806. Combined with the clinical significance, we finally input the following four variables into the model: gender, number of PLNs, Breslow thickness and ulceration. The selected factors were analyzed by MSS survival analysis, and the results are shown in Figure S4.

### 3.4 Nomogram construction and validation

**FIGURE 2:**
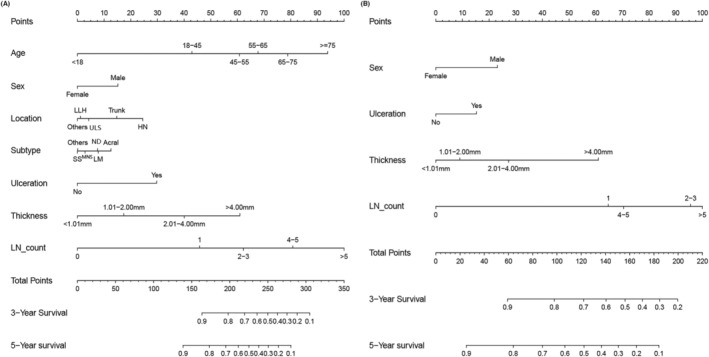
(A) Nomogram predicting 3‐ and 5‐year survival for patients who underwent excisional surgery with NMCM. Location: Acral, acral lentiginous melanoma; HN, head and neck; LLH, lower limb and hip; ULS, upper limb and shoulder. Subtype: LM, Lentigo malignant; MNS, Melanoma not specified; ND, Nodular; SS, Superficial spreading. (B) Nomogram predicting 3‐ and 5‐year survival for patients underwent amputation with NMCM. NMCM, non‐metastasis cutaneous melanoma.

**FIGURE 3:**
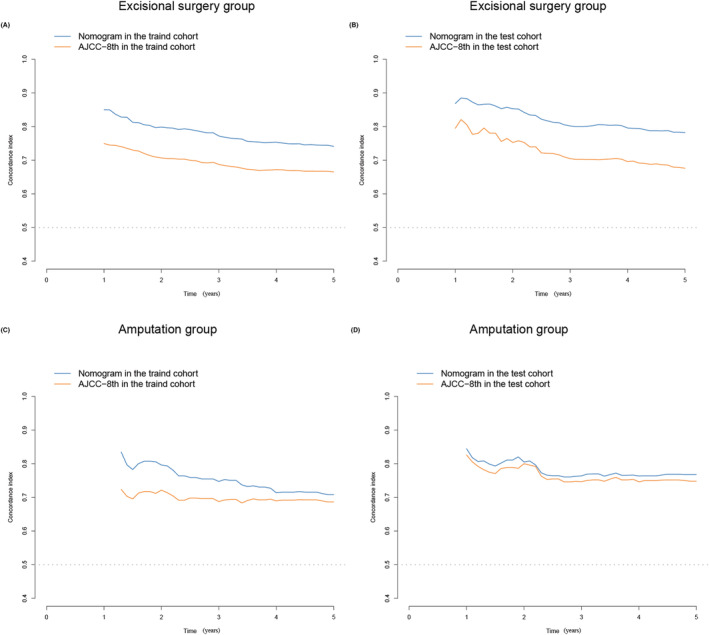
Time‐dependent C‐index of the current models and the AJCC 8th staging system. (A) Time‐dependent C‐index in the trained bootstrap verification cohort of the excisional surgery group; (B) Time‐dependent C‐index in the test cohort of the excisional surgery group; (C) Time‐dependent C‐index in the trained bootstrap verification cohort of the amputation group; (D) Time‐dependent C‐index in the test cohort of amputation group. AJCC, American Joint Committee on Cancer.

**FIGURE 4:**
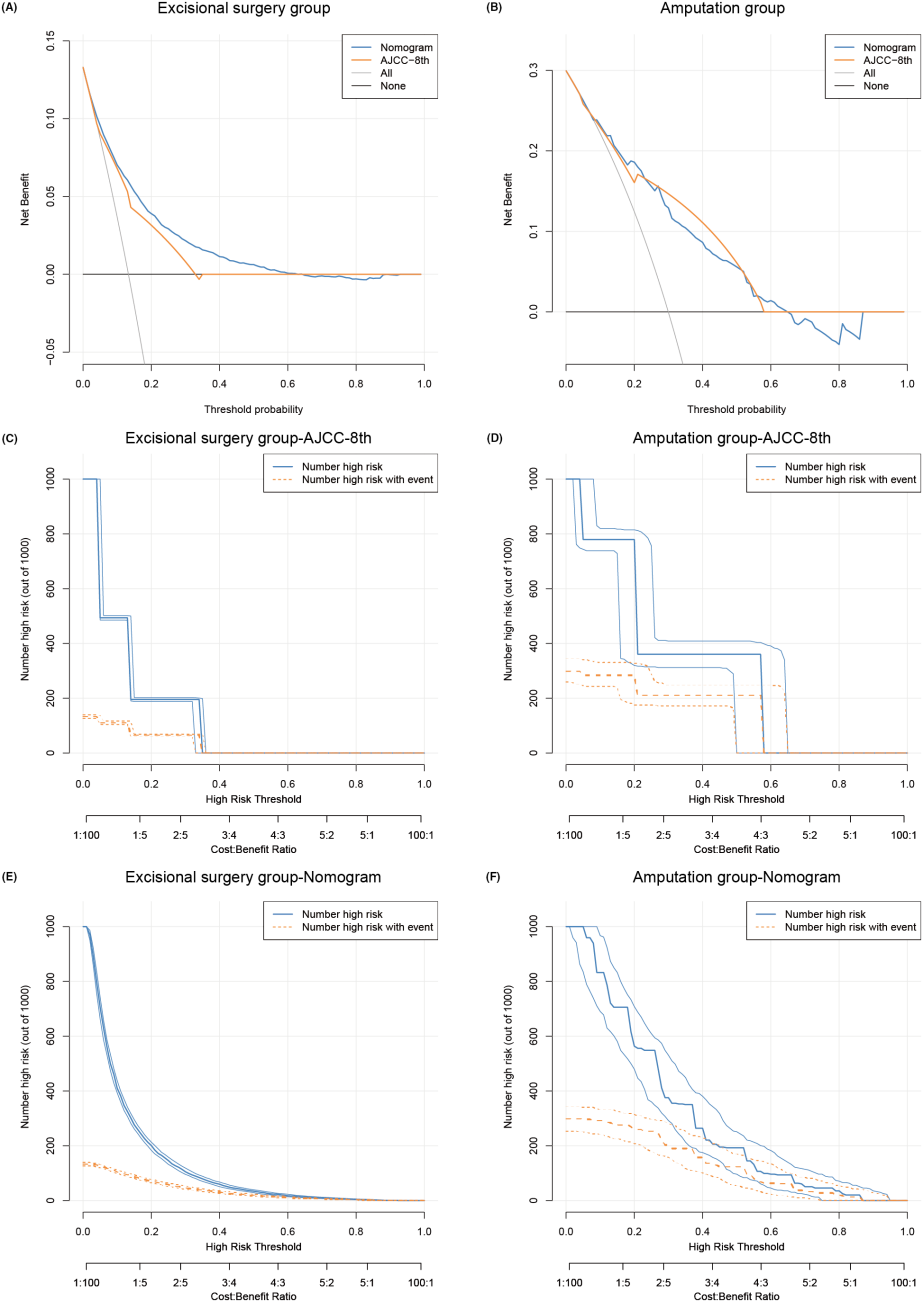
DCA and clinical influence curves of the current models and the AJCC staging system predicting MSS. (A) DCA of the nomogram for the excisional surgery group and the AJCC staging system; (B) DCA of the nomogram for the amputation group and the AJCC staging system; (C) Clinical influence curves of the AJCC 8th staging system for the excisional surgery group; (D) Clinical influence curves of the AJCC 8th staging system for the amputation group; (E) Clinical influence curves of the nomogram for the excisional surgery group; (F) Clinical influence curves of the nomogram for the amputation group. AJCC, American Joint Committee on Cancer; DCA, decision curve analysis; MSS, melanoma‐specific survival.

Based on the predictive variables selected in the previous section, we established two nomograms to predict the 3‐ and 5‐year MSS of NMCM patients. The nomograms of the patient group undergoing excisional surgery and the patient group undergoing amputation are shown in Figure 2A,B, respectively. To verify the accuracy of the nomograms, we measured the C‐index of the model. The results showed that the C‐index of the excisional surgery group was 0.818, and that of the amputation group was 0.806. The C‐index of the model verified by our hospital data was 0.763 and 0.731, respectively. In Figure S5, we made the receiver operating characteristic(ROC) curve of both nomograms, which showed that the AUC values of 3‐year and 5‐year MSS in the excisional surgery group were 0.856 and 0.844, respectively, and those in the amputation group were 0.808 and 0.844, respectively. To further observe the predictive ability of the model at different time points, we divided the data into the training group and internal verification group and used the dynamic C‐index to compare the predictive ability of the nomogram model with the AJCC 8th staging system. As shown in Figure 3, the blue line representing the nomogram model was always entrenched above the yellow line representing the AJCC 8th stage, which means nomograms always have good prediction accuracy in the prediction of 0–5 year MSS. To evaluate the clinical usefulness of the nomograms, we measured their net benefit using the DCA curve and compared it with the AJCC 8th staging system. The results showed that the nomograms had broad and practical threshold probabilities (Figure 4A,B). The clinical impact curve showed that the number of nomogram blue solid lines classified as positive (high risk) is closer to that of yellow dotted lines compared with the AJCC 8th staging system (Figure 4C–F); that is, the clinical influence is more accurate.

### 3.5 Development of two web calculators based on nomograms

To facilitate clinical application, we set up two web calculators based on the shinyapp.io platform. The links to the web pages are as follows: for the excisional surgery group: https://surgerymelanoma.shinyapps.io/Excisionalsurgery/; for the amputation group: https://surgerymelanoma.shinyapps.io/Amputation/. Clinicians can quickly obtain access to the MSS values of patients by simply selecting clinical variables. The application software can also generate a graphical summary of survival probabilities, a graphical representation of estimated survival probabilities during follow‐up, and a digital summary table.

## 4 DISCUSSION

Surgical removal of melanoma is a crucial treatment for CM patients without distant metastasis. However, the choice of margin width for CM excisions is controversial.^5^, ^6^, ^9^, ^10^ It remains debatable whether the reduction in survival after amputation is due to the underlying condition of the patient or to the negative effects of the amputation itself (including on hemodynamics and mental health).^11^, ^12^ In this study, based on a large amount of data in the SEER database, we collected NMCM cases with different widths of excision margins for surgery and amputation. We found survival rates were similar in the excision margin 1–2 cm group and the excision margin >2 cm group after correcting for other influencing factors. This means a 1–2 cm margin is non‐inferior to a > 2 cm margin for NMCM patients' survival, consistent with several published results.^6^, ^9^, ^10^ For CM without distant metastases, a smaller margin of 1–2 cm can help preserve limb integrity and function as much as possible. The fact that the amputation group continued to have a more malignant prognosis after correcting for other factors further suggests that we should be more cautious about the decision to amputate. Major limb amputations are often used when limb salvage strategies, such as isolated limb infusion, isolated limb perfusion, and local treatments, including intrafocal injection and radiotherapy, are exhausted.^11^ Patients who undergo radical amputation can achieve 5‐year survival rate at about 15%–30%.^7^, ^8^ Causes of death after amputation include melanoma metastasis, recurrence, complications, and the patient's health problems.^13^ Furthermore, in addition to the possible residual sequelae of pain or vascular and neurological damage from amputation,^14^ the patient's psychological distress should be taken into account in clinical decision‐making.^15^ Although nowadays amputation is usually used as a last resort and is rarely indicated (<1%).^7^, ^13^ In advanced care centers with more critically ill patients, such as Chinese tertiary hospitals, we have observed a nonnegligible rate of amputation. In our hospital, 15.69% of patients admitted with CM in the last decade had their limbs amputated (Table S1). Predictive models, especially for amputation, can help to assess the prognosis of these patients.

Based on the results of the Cox survival analysis, we next divided the patients into two cohorts (excisional surgery group and amputation group) and analyzed the prognostic factors for patient survival separately. The main prognostic factors of the nomogram in our excisional surgery group included gender, age, pathological subtype, number of PLNs, ulceration, primary tumor location, and Breslow thickness (mm). These prognosis factors also have been demonstrated in previous studies.^16^, ^17^, ^18^, ^19^ Women have been demonstrated to have a survival advantage in melanoma, which may be explained by the fact that women pay more attention to their bodies, have less ultraviolet exposure to sunlight and have more estrogen effect on melanoma growth and metastasis.^20^, ^21^, ^22^ A negative correlation between survival and age has also been reported in other studies and is associated with more aggressive tumors caused by a decline in the ability of the immune system.^23^, ^24^ Our data show that among the pathological subtypes, the superficial spreading subtype has the best prognosis. This may be due to its superficial spreading growth characteristics, which are less likely to reach skin vessels and lymph nodes.^25^ In contrast, nodular and acantholytic melanomas have lower survival rates.^26^, ^27^ The relationship between the number of positive lymph nodes and the survival rate has also been reported in many studies.^28^, ^29^ According to the number of positive lymph nodes, NMCM patients were divided into four subgroups according to the number of positive lymph nodes (0, 1, 2–3, and ≥4). In our study, we found that in the group with multiple lymph nodes, the number 5 was also a cut‐off (using the R language cut‐off packet) (Table 3).

Many studies have confirmed the importance of ulceration in the prognosis of melanoma and in reducing the disease‐free survival and survival of patients.^30^, ^31^, ^32^ In this study, the location of the primary tumor was also used as a predictor, and its prognostic effect was also verified by other studies.^33^, ^34^ Lesions with thicker Breslow thickness can represent more advanced tumors with more intrinsic biological invasiveness than those with radial growth alone.

The main prognostic factors of the nomogram in our amputation surgery group only included gender, number of PLNs, ulceration, and Breslow thickness (mm). Chi‐square tests indicated that pathological subtype and location could influence the decision on surgical approach (*p* < 0.001) (Table 1). However, it is noteworthy that these were not factors that influenced survival after treatment (Table 2). A typical example is ALM: patients with ALM are more likely to undergo amputation because of its prevalence at the extremity (29.70% [amputation group] vs 3.42% [excisional surgery group]) (Table 1). However, the prognosis of ALM after amputation was not significantly different from other pathological subtypes, and therefore ALM could not be a prognostic factor (Table 2), in agreement with the results of previous studies.^34^, ^35^, ^36^

Finally, given that the prognostic factors differed so much between the excisional surgery and amputation groups, we constructed nomograms to predict MSS by combining other meaningful clinical data variables, respectively. They showed good accuracy and utility in the validation of internal data and external data from Affiliated Drum Tower Hospital, Medical School of Nanjing University, with good predictive performance even compared with the AJCC 8th staging system. The web version was also created for more convenient use.

As mentioned above, our research has the following characteristics that make our research prominent and vital: (1) We developed two simple‐to‐use nomograms and corresponding online web calculators for the excisional surgery and amputation groups. Online tools not only allow clinicians to generate MSS estimates but also graphically display estimated survival probabilities and digital summary tables of survival estimates during follow‐up; (2) Both the training group and the verification group have a large amount of data to verify the accuracy and practicability of the models; (3) Good reliability was obtained by using the data of our hospital for external verification; (4) We screened and analyzed prognostic factors separately for the excisional surgery and amputation groups; (5) Our study, based on the Cox survival analysis of large‐amount SEER data, provides evidence that surgical margins 1–2 cm or >2 cm have no significant impact on survival in NMCM patients, while suggesting using amputation for CM with more caution.

There are some limitations to this study. First, as a retrospective study, it has inherent shortcomings. Although the sample size was large, it still needs further verification. Also, although we used a dataset collected at our hospital over the last decade, the data were insufficient and needed to be validated with data from multiple healthcare institutions. Second, not enough details of systematic treatment were collected. Future studies are needed to clarify the impact of these factors on patient survival. A more comprehensive synthesis of these factors may improve the predictive ability of our model. Third, there is no specific amputation location and details recorded in the SEER database. More detailed clinical studies are needed to further explain the prognosis of different amputations in the future.

In conclusion, we observed comparable survival rates for individuals with NMCM regardless of the extent of the resection margin, but considerably worse survival rates for those who required amputation by comparing the data from the SEER database. We analyzed the prognostic factors of the excisional surgery and the amputation group separately and established two different prognostic nomograms according to clinically significant variables. Two web calculators were established based on our nomograms so that the prognosis graph could be outputted easily online for facilitating clinical use. After internal and external verification, our nomograms showed good accuracy and clinical practicability.

## AUTHOR CONTRIBUTIONS

**Shiqi Wang:** Conceptualization (equal); data curation (equal); writing – original draft (equal). **Yuedong Chen:** Conceptualization (equal); data curation (equal); writing – original draft (equal). **Jiawei Sun:** Data curation (equal). **Ran Mo:** Conceptualization (equal); data curation (equal). **Qian Tan:** Supervision (equal); writing – review and editing (equal).

## FUNDING INFORMATION

None.

## CONFLICT OF INTEREST STATEMENT

The authors have no conflicts of interest to declare.

## ETHICS STATEMENT

The authors are accountable for all aspects of the work in ensuring that questions related to the accuracy or integrity of any part of the work are appropriately investigated and resolved. All procedures performed in this study involving human participants were in accordance with the Declaration of Helsinki (as revised in 2013). This study was reviewed and approved by the Ethics Committee of Nanjing Drum Tower Hospital, the Affiliated Hospital of Nanjing University Medical School (approval no. 2017–175‐01), *and individual consent for this retrospective analysis was waived*.

## Supporting information


Figure S1.

Figure S2.

Figure S3.

Figure S4.

Figure S5.

Table S1:

## Data Availability

The datasets used and analyzed during the current study are available from the Surveillance, Epidemiology, and End Results (SEER) database (http://seer.cancer.gov/data/sample‐dua.html).
