# Supplementary figures and images for: Development and validation of two online dynamic nomograms for patients with non‐distant metastatic cutaneous melanoma based on surgical approaches
Source: Cancer Med. 2023 Aug 18;12(18):18479–90. doi: 10.1002/cam4.6448 (PMC10557963; doi:10.1002/cam4.6448)

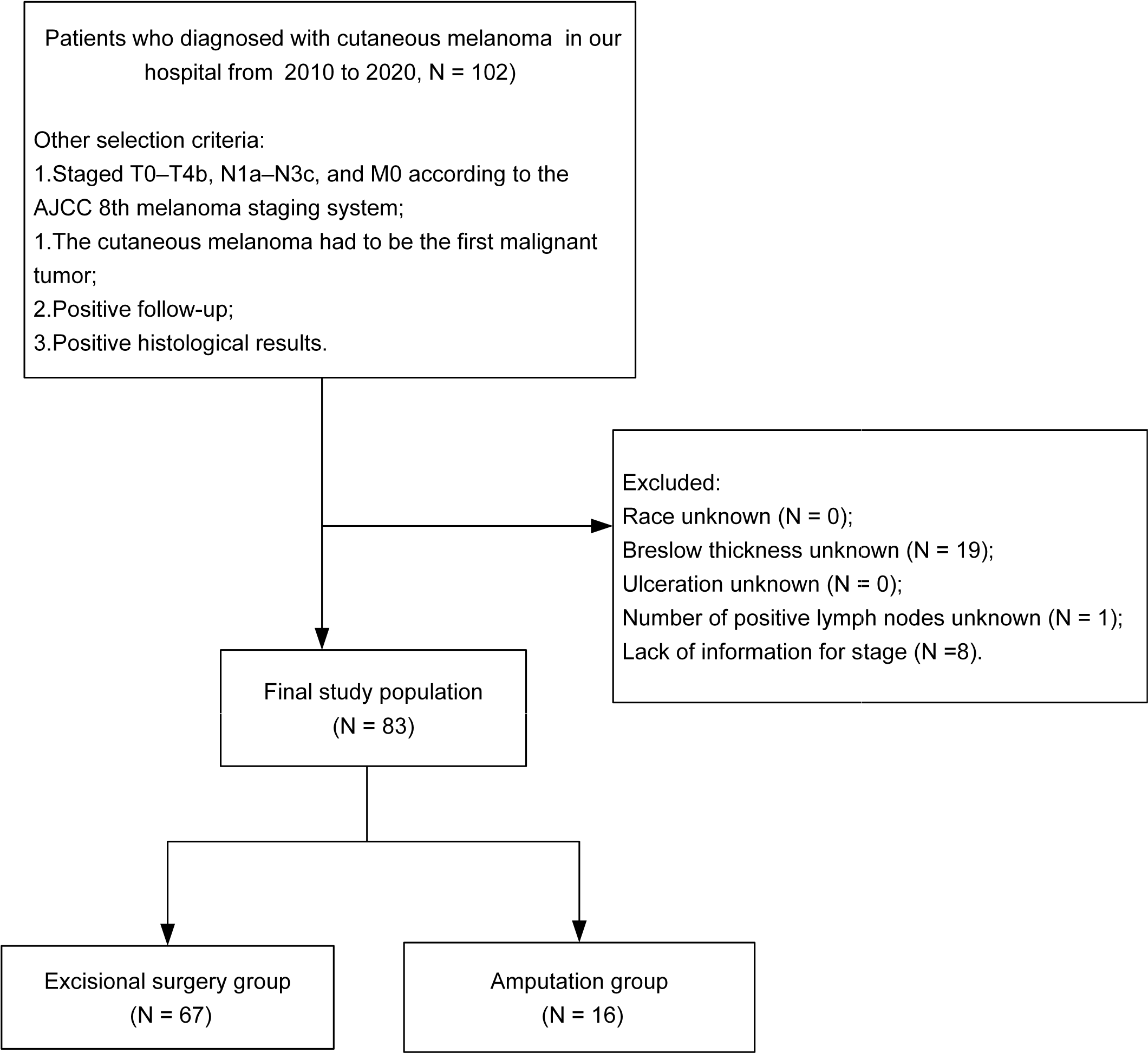

Supplement: Supplementary file 1 — Figure S1. [file CAM4-12-18479-s006.tif]

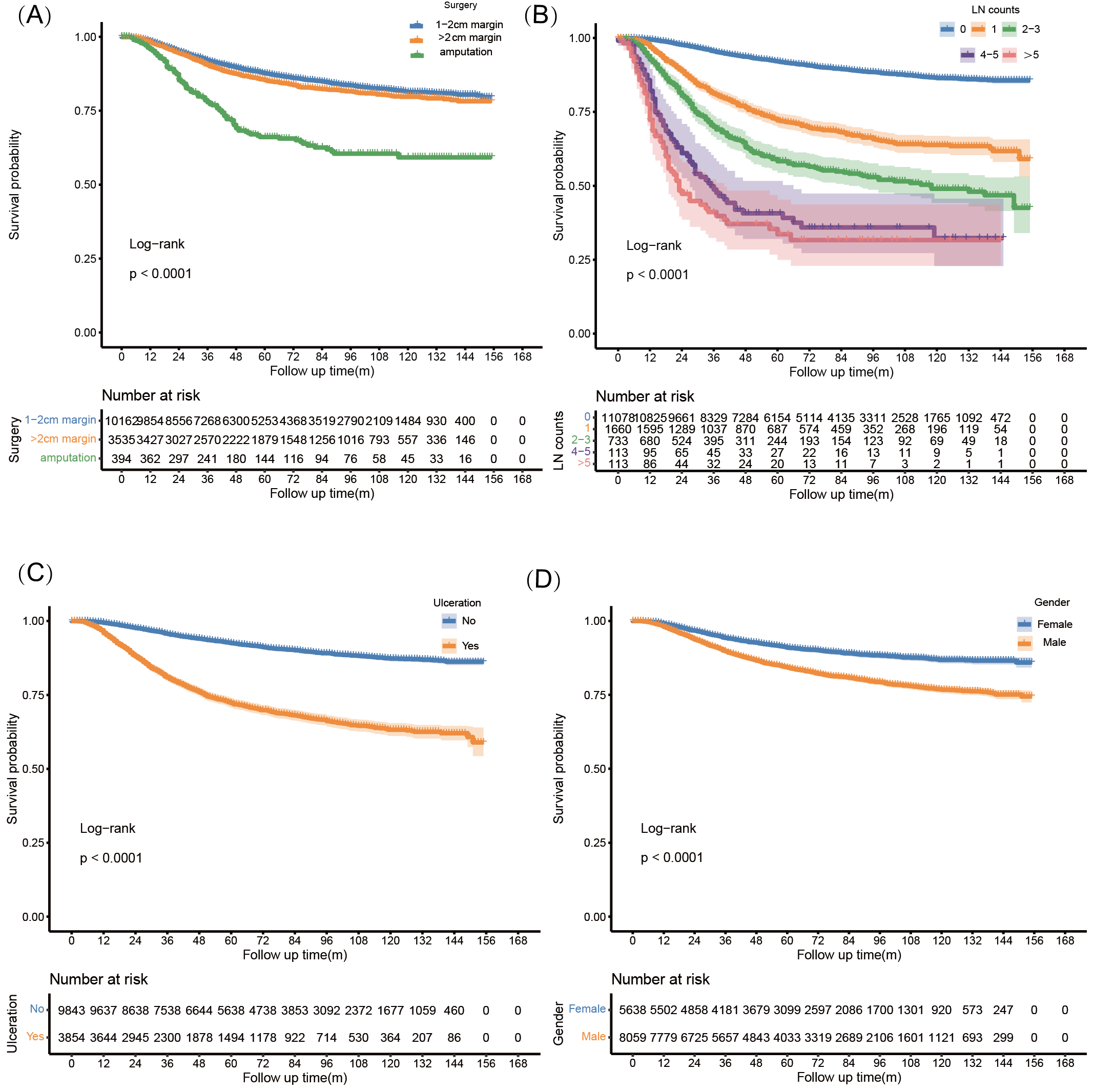

Supplement: Supplementary file 2 — Figure S2. [file CAM4-12-18479-s002.tif]

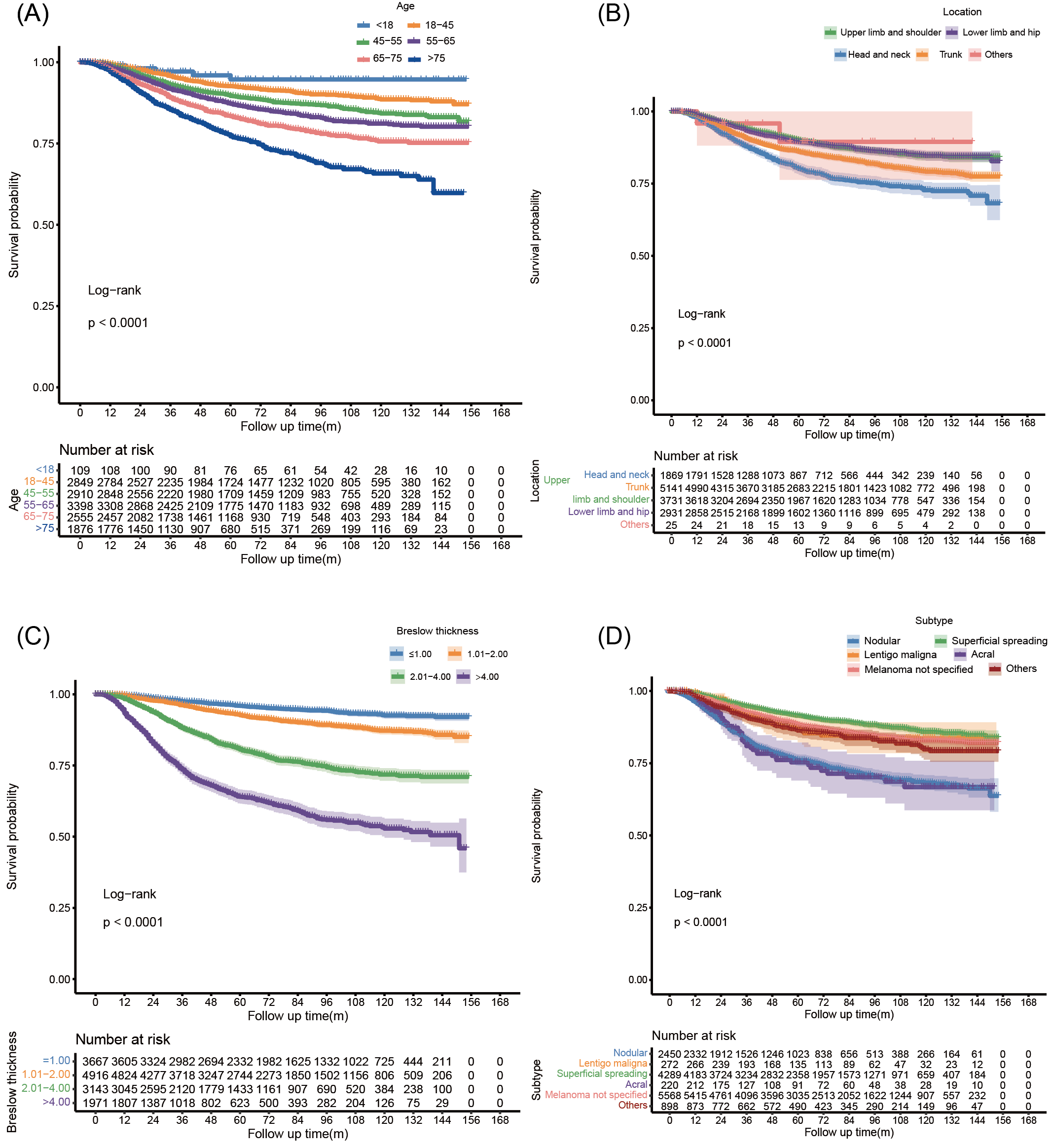

Supplement: Supplementary file 3 — Figure S3. [file CAM4-12-18479-s003.tif]

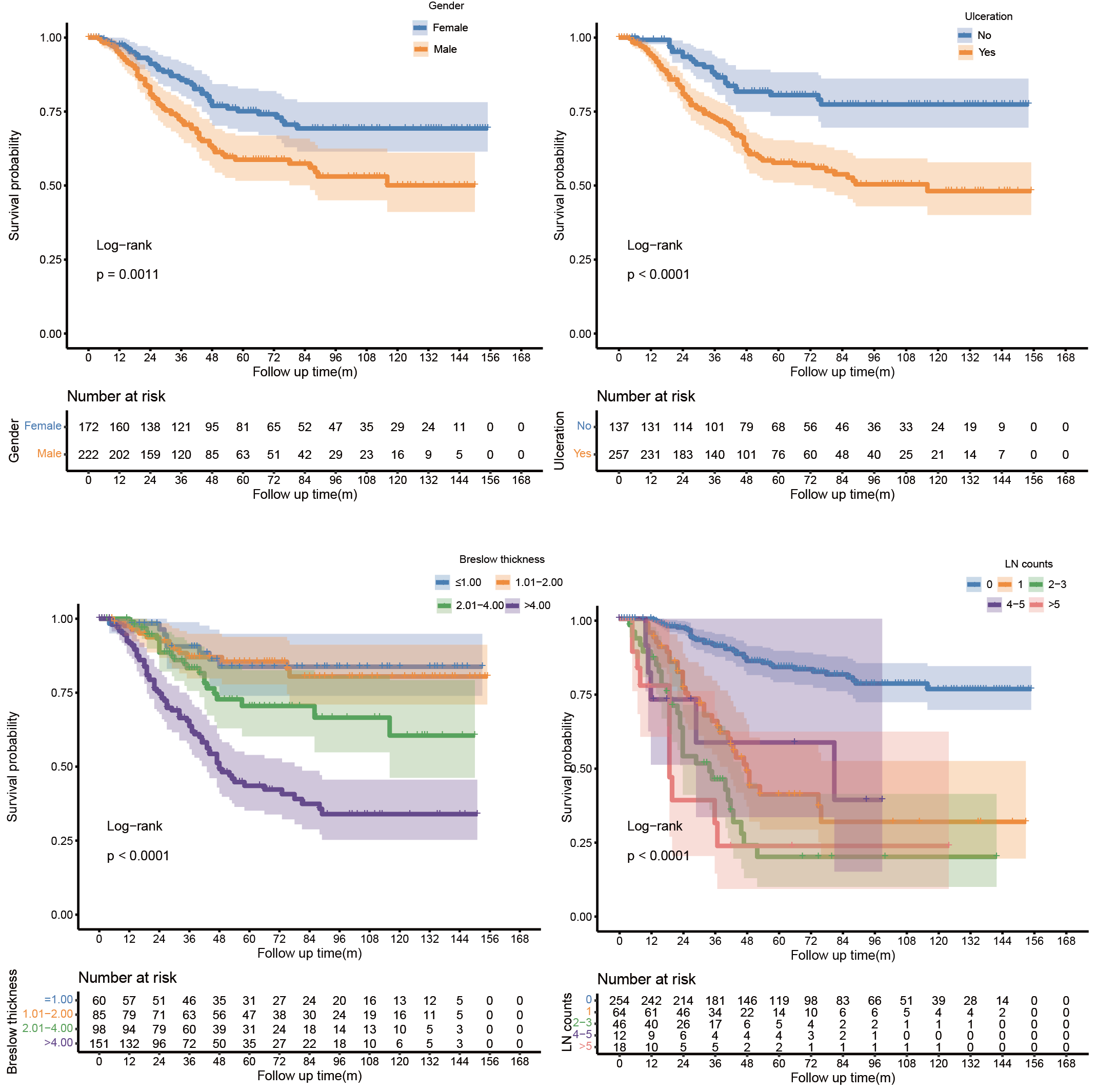

Supplement: Supplementary file 4 — Figure S4. [file CAM4-12-18479-s004.tif]

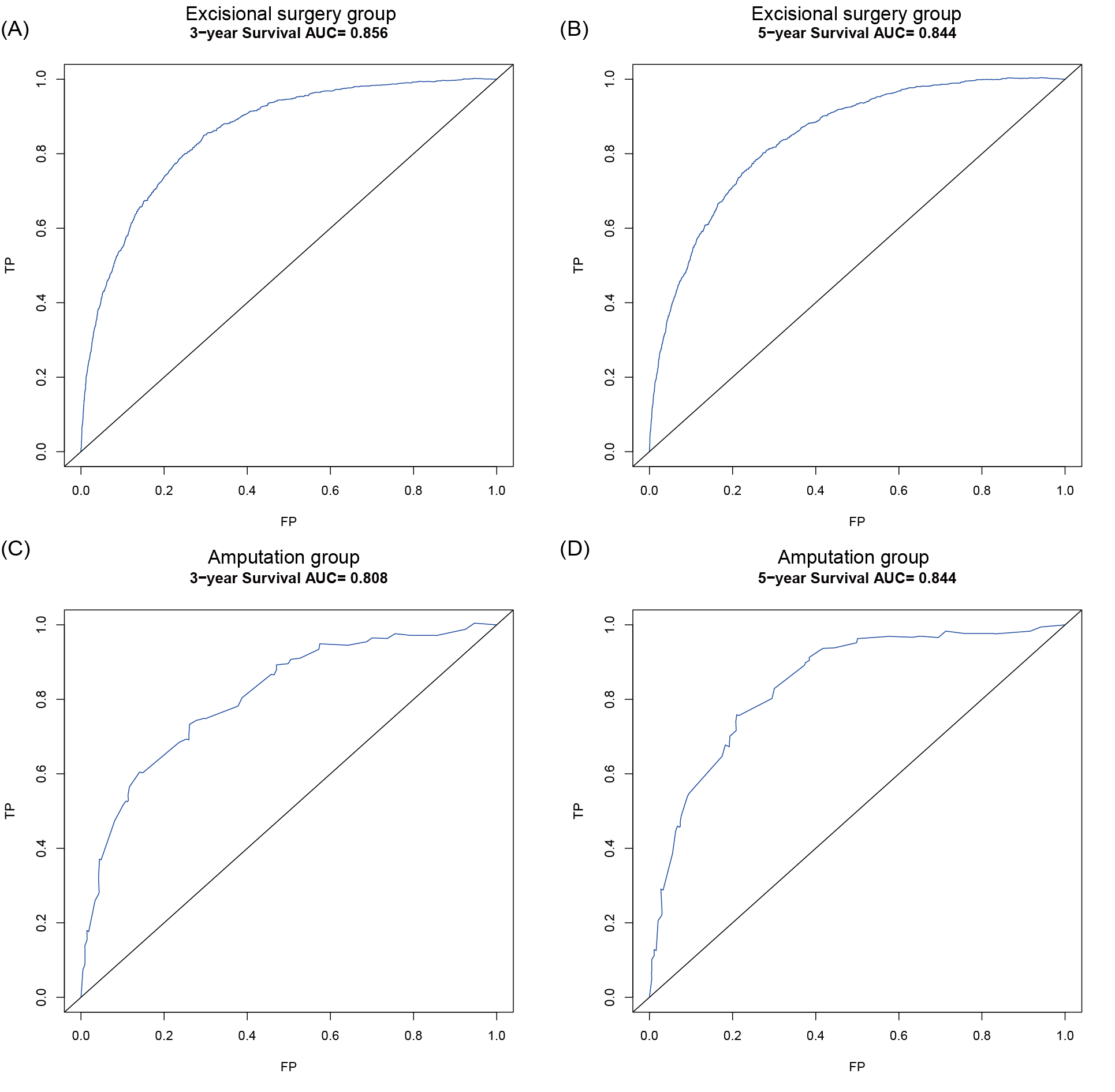

Supplement: Supplementary file 5 — Figure S5. [file CAM4-12-18479-s005.tif]
