# Supplementary material for: Development and validation of two online dynamic nomograms for patients with non‐distant metastatic cutaneous melanoma based on surgical approaches
Source: Cancer Med. 2023 Aug 18;12(18):18479–90. doi: 10.1002/cam4.6448 (PMC10557963; doi:10.1002/cam4.6448)
Supplement: Supplementary file 6 — Table S1: [file CAM4-12-18479-s001.docx]

Supplementary Table S1: Baseline clinicopathologic characteristics of a Chinese cohort according to different surgical methods. MSS, melanoma-specific survival.

|  | n(%) |  |  |  |
| --- | --- | --- | --- | --- |
| Variables | Excisional surgery (n=67,80.72%) | Amputation (n=16,19.28%) | Total(n=83) | *p*-value^#^ |
| **Sex** |  |  |  | 0.391 |
| Female | 37 (55.22) | 6 (37.50) | 43 (51.81) |  |
| Male | 30 (44.78) | 10 (62.50) | 40 (48.19) |  |
| **Age** | 63.01±12.70 | 63.06±13.80 | 63.02±12.83 |  |
| **Age** |  |  |  | 0.783 |
| <18 | 6 (8.96) | 2 (12.50) | 8 (9.64) |  |
| 18-44 | 8 (11.94) | 2 (12.50) | 10 (12.05) |  |
| 45-54 | 20 (29.85) | 6 (37.50) | 26 (31.33) |  |
| 55-64 | 20 (29.85) | 2 (12.50) | 22 (26.51) |  |
| 65-75 | 12 (17.91) | 4 (25.00) | 16 (19.28) |  |
| ≥75 | 1 (1.49) | 0 (0.00) | 1 (1.20) |  |
| **Location** |  |  |  | 0.180 |
| Head and neck | 5 (7.46) | 0 (0.00) | 1888 (6.02) |  |
| Trunk | 5 (7.46) | 0 (0.00) | 5144 (6.02) |  |
| Upper limb and shoulder | 4 (5.97) | 3 (18.75) | 3856 (8.43) |  |
| Lower limb and hip | 53 (79.10) | 13 (81.25) | 3177 (79.52) |  |
| **Subtype of melanoma** |  |  |  | 0.901 |
| Nodular melanoma | 18 (26.87) | 5 (31.25) | 23 (27.71) |  |
| Superficial spreading melanoma | 3 (4.48) | 0 (0.00) | 3 (3.61) |  |
| Acral lentiginous melanoma | 21 (31.34) | 5 (31.25) | 26 (31.33) |  |
| Melanoma not specified | 24 (35.82) | 6 (37.50) | 30 (36.14) |  |
| Others | 1 (1.49) | 0 (40.36) | 1 (1.20) |  |
| **Lymph node count** |  |  |  | 1 |
| 0 | 64 (95.52) | 15 (93.75) | 79 (95.18) |  |
| 1 | 3 (4.48) | 1 (6.25) | 4 (4.82) |  |
| **Ulceration** |  |  |  | 1 |
| No | 45 (6.716) | 11 (68.75) | 131 (67.47) |  |
| Yes | 22 (32.84) | 5 (31.25) | 9980 (32.53) |  |
| **Breslow thickness (Mean ± SD) (mm)** | 4.00±4.76 | 6.90±11.71 | 4.60±6.68 |  |
| **Breslow thickness (mm)** |  |  |  | 0.136 |
| ≤1.00 | 11 (16.42) | 2 (12.50) | 13 (15.66) |  |
| 1.01-2.00 | 16 (23.88) | 3 (18.75) | 19 (22.89) |  |
| 2.01-4.00 | 22 (32.84) | 2 (12.50) | 24 (28.92) |  |
| >4.00 | 18 (26.87) | 9 (56.25) | 27 (32.53) |  |
| **MSS** |  |  |  |  |
| Alive | 63 (94.03) | 13 (81.25) | 76 (91.57) | 0.249 |
| Dead | 4 (5.97) | 3 (18.75) | 7 (8.43) |  |
